# Necrology

**Published:** 1896-12

**Authors:** 


### NECROLOGY.

The death of Edwin Willits, A.M., LL.D., Lecturer on Medical Jurisprudence at the National Veterinary College, Washington, D. C., removes a strong friend and supporter of this school, and ends a well-lived and useful life, at a time when the value of his long years of faithful service in advancing agriculture in the broadest acceptation of that term was of the greatest worth and most sorely needed in the history of our country. Dr. Willits had filled well the posts of President of the Agricultural College of Michigan, thrice representing his home district in Michigan in the lower branch of Congress, and Assistant Secretary of Agriculture under Hon. Jeremiah Rusk, and continued for a time under Secretary Morton. His interest in the advancement of veterinary science was a matter of great pride, and his recognition of its value to the prosperous growth of agriculture such as to make his value as a teacher of much importance.

